# Facile In Situ Fabrication of Nanostructured Graphene–CuO Hybrid with Hydrogen Sulfide Removal Capacity

**DOI:** 10.1007/s40820-016-0090-8

**Published:** 2016-03-23

**Authors:** Sunil P. Lonkar, Vishnu V. Pillai, Samuel Stephen, Ahmed Abdala, Vikas Mittal

**Affiliations:** Department of Chemical Engineering, The Petroleum Institute, Abu Dhabi, United Arab Emirates

**Keywords:** CuO/graphene, Adsorption, Breakthrough capacity, Hydrogen sulfide, Thermal stability, In situ synthesis

## Abstract

A simple and scalable synthetic approach for one-step synthesis of graphene–CuO (TRGC) nanocomposite by an in situ thermo-annealing method has been developed. Using graphene oxide (GO) and copper hydroxide as a precursors reagent, the reduction of GO and the uniform deposition of in situ formed CuO nanoparticles on graphene was simultaneously achieved. The method employed no solvents, toxic-reducing agents, or organic modifiers. The resulting nanostructured hybrid exhibited improved H_2_S sorption capacity of 1.5 mmol H_2_S/g-sorbent (3 g S/100 g-sorbent). Due to its highly dispersed sub-20 nm CuO nanoparticles and large specific surface area, TRGC nanocomposite exhibits tremendous potential for energy and environment applications.

## Introduction

The combination of multidimensional nanomaterials often leads to the formation of hierarchical and multifunctional materials that combine the advantages of each component, thus, resulting in exceptional properties. Recently, composites of graphene with various inorganic nanostructures including copper oxide (CuO) have attracted a great deal of interest due to synergistic combination of properties and potential applications [1–3]. In general, it is believed that incorporation of multidimensional inorganic particles may prevent the aggregation of graphene sheets with higher surface area and pore volume. Similarly, the graphene sheets can effectively stabilize inorganic nanoparticles to prevent their aggregation, and the properties of the nanoparticles could be enhanced through anchoring them onto graphene sheets [4–6]. Graphene, the 2-D *sp* ^2^ network of carbon atoms possesses several intriguing and peculiar properties such as high charge mobility [100,000 cm^2^ (V s)^−1^], high surface area (2630 m^2^ g^−1^), thermal conductivity (2000–5000 Wm K^−1^), and optical properties [7]. On the other hand, CuO, an important *p*-type transition-metal oxide with a narrow band gap (*E*_g_ = 1.2 eV) and excellent chemical stabilities, has been investigated extensively for active anode materials, superconductors, sensors, and heterogeneous catalysts. It is also a promising material for fabricating solar cells, due to its photoconductive and photochemical properties [8–10]. Hence, hybridization of these two materials leads to nanohybrids of graphene and CuO with outstanding properties suitable for a variety of applications such as sensors [11–13], photocatalysis [14], water treatment [15], energy storage [16, 17], etc.

Several approaches have been proposed for preparing nanostructured composites of graphene with CuO which mostly involve either deposition of CuO nanoparticle on GO sheets followed by the reduction of GO or first to reduce GO sheets and then deposit or grow nanocrystals on the graphene sheets [18]. These methods often use toxic or hazardous reducing agents such as hydrazine [19], sodium borohydride, and ammonia [20] for GO reduction. Moderate-to-severe synthesis conditions including different unfavorable solvents or microwave energy under variable pH, temperature, and pressures were employed. The most common methods for graphene/CuO synthesis include hydrothermal [15, 21, 22], sol–gel, microwave [17, 23, 24], sonochemical [25], and surfactant-assisted synthesis [26]. These methods need specific selection of appropriate synthesis conditions and suitable surfactants. Hence, there is a need to develop a greener, scalable, and facile route for the direct synthesis of nanostructured graphene/CuO composites. So far, there is no report on direct solvent-free synthesis of in situ born CuO nanoparticles on reduced graphene oxides sheets without addition of toxic-reducing agent. Therefore, a cost-effective, environmentally friendly, and scalable production of nanostructured graphene/CuO composites can help to enhance ever-growing applications of such materials further. Herein, for the first time, the preparation of graphene/CuO nanostructured composite via in situ process under mechano-thermal conditions using copper hydroxide and graphite oxide reagents is reported. No solvents, toxic-reducing agents, or surfactants are used. This simple and environmentally benign method is easily scalable for large scale production of high-quality graphene/CuO composite suitable for a wide range of applications.

Hydrogen sulfide (H_2_S) is one of the most common and undesirable sulfur component often found in natural gas, syngas, biogas, and other industrial gases [27, 28]. Due of its high toxicity, offensive odor, and acidic nature, H_2_S can pose serious threats to health and environment. Moreover, trace amount of H_2_S can cause catalyst poisoning and pipeline corrosion [29]. Its oxidation in the atmosphere to SO_2_ results in an acid rain formation. Therefore, development of the new adsorbent materials with excellent desulfurization performance is critically needed. Owing to its thermal stability as well as favorable thermodynamics in sulfidization reaction, copper oxide is considered as a very effective metal oxide sorbent for the removal of H_2_S from various gas streams [30, 31]. Additionally, copper-based sorbent does not suffer from metal volatility problems like other metallic sorbents. Copper oxide reacts with hydrogen sulfide to form the insoluble copper sulfide [32]. Hybridization of H_2_S active CuO at nanoscale with high surface area support like graphene can further enhance the overall adsorption capacity of H_2_S. Thus, in this case, degree of oxygen functional groups and surface area of graphene in conjunction with particle size and distribution of metal oxide nanoparticles would be conducive for low-temperature H_2_S adsorption [33]. Moreover, the graphene/CuO hybrid materials have not been explored as a sorbents for H_2_S removal. Hence, in the present work, the effect of the sub-20 nm CuO supported onto high surface area graphene is investigated as a sorbent for H_2_S removal.

## Experimental

### Materials

Graphite powder (Sigma-Aldrich, 10 mesh), sulfuric acid (Sigma-Aldrich, ACS reagent, 95.0–98.0 %), hydrochloric acid (Sigma-Aldrich, ACS reagent, 37 %), potassium permanganate (Fischer Scientific, ≥99 %), hydrogen peroxide (Sigma-Aldrich, 30 wt% in H_2_O), sodium hydroxide (Sigma-Aldrich, ACS reagent, ≥97.0 %), copper nitrate dehydrate (Sigma-Aldrich, ACS reagent, ≥98 %), and phosphoric acid (Sigma-Aldrich, ACS reagent, ≥85 wt% in H_2_O) were used. Copper hydroxide was prepared by procedure presented elsewhere [34].

### Synthesis of Graphite Oxide (GO)

Graphite oxide was prepared from natural graphite by using improved synthesis proposed by Tour et al. [35]. In brief, the mixture of concentrated sulfuric acid (270 mL) and phosphoric acid (33 mL) was added to a 5 l Erlenmeyer flask placed in an ice bath. About 5 g of natural flake graphite (10 mesh) was dispersed in the cold sulfuric acid with an overhead stirrer. Subsequently, 2.7 g of KMnO_4_ was added slowly over 1520 min, and the resulting one-pot mixture was stirred for 72 h at room temperature to allow the oxidation of graphite. The color of the mixture changed from dark purple-green to dark brown. Later, about 35 % hydrogen peroxide (H_2_O_2_) solution was added to terminate the oxidation process, and the color of the mixture changed to bright yellow, indicating a high oxidation level of graphite. The as-synthesized graphite oxide was suspended in water containing 1 M dilute hydrochloric acid to obtain a yellow–brown dispersion, which was subjected to repeat washing with de-ionized water until a pH of 4–5 was achieved. To ensure complete removal of the residual salts and acids, dialysis process was used.

### Preparation of Graphene/CuO Nanohybrid (TRGC)

Aqueous dispersions of GO (200 mg) and stoichiometric quantity of copper hydroxide were prepared under ultra-sonication for 30 min and rapidly mixed at room temperature in a round-bottomed flask followed by stirring. The resulting homogenous mixture was freeze-dried at −90 °C to obtain GO–Cu salt composite. Further, the composites were thermally annealed in a tube furnace at 400 °C for 2 h under argon atmosphere with a heating rate of 5 °C min^−1^ to finally obtain graphene/CuO composite. A color change from light brown to black was also noticed. A stoichiometric quantity of copper hydroxide was used in order to obtain TRGC composite with 10 wt% CuO loading. For comparison, thermally reduced graphene oxide (TRG) and copper oxide nanoparticles were synthesized under similar conditions and abbreviated as TRG and CuO, respectively.

### Characterization

The TRGC nanohybrid was characterized by X-ray diffraction (XRD), X-ray photoelectron spectroscopy (XPS), transmission electron microscopy (TEM), scanning electron microscopy (SEM), Raman spectroscopy, thermogravimetric analysis (TGA), and N_2_ physisorption. XRD was performed using Cu*K*α radiation (X’Pert Pro X-Ray diffractometer from Philips) at angle range (2*θ*/5–60°). The XPS measurements were performed on an SSX-100 system (Surface Science Laboratories, Inc.) equipped with a monochromated Al *K*_α_ X-ray source, a hemispherical sector analyzer (HSA) and a resistive anode detector. TEM analysis was performed using FEI Tecnai G20 with 0.11 nm point resolution and operated at 200 kV using Gatan digital camera. SEM (1540 XB Zeiss) coupled with energy-dispersive X-ray analysis (EDX) was used to determine the structure of the nanohybrids. LabRAM HR (Horiba Scientific) was used to obtain Raman spectra. Typically, a 50× objective was used with 633 nm excitation line. TGA was carried out by using Discovery TGA (TA instruments) in the temperature range from 50 to 800 °C at a ramp rate of 10 °C in an air atmosphere (30 mL min^−1^). N_2_ physisorption was carried out at liquid N_2_ temperature with a Micromeritics ASPS 2010 analyzer to examine the porosity and surface area of the sample. The sample was pre-treated at 100 °C in a high vacuum for 24 h before N_2_ adsorption.

### H_2_S Sorption Studies

**Fig. 1:**
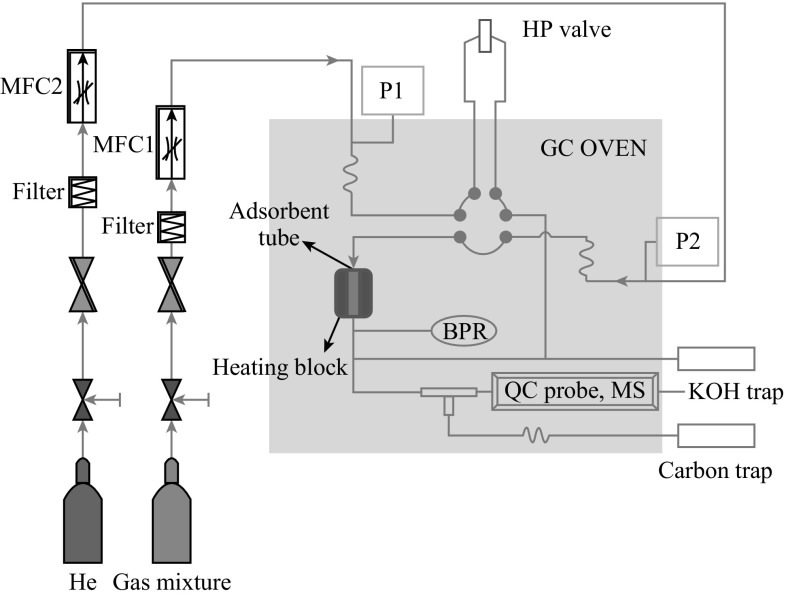
Schematic of the fixed-bed flow system for H_2_S adsorption measurements

The H_2_S sorption experiments were carried out at room temperature (30 °C) and 290 psig pressure. The sorption tube was made of glass with an outer diameter of 8 mm and the height of 20 mm, into which ~0.5 g of the adsorbent was packed (Fig. 1). For adsorption, a model gas mixture containing (99.4 % of CH_4_, 0.41 % of CO_2_, and 0.15 % of H_2_S) was passed through the adsorption cell with a flow rate of 40 mL min^−1^. The gas mixture was delivered through the books mass flow controller at fixed flow rate. The analysis of the breakthrough gas was performed using a quadrupole mass spectrometer. Helium was used as marker gas. The breakthrough and saturation capacity [denoted as Cap (BT), mmol g^−1^, STP] for H_2_S was calculated according to the following equation [36]:

1 $${\text{Cap}}\left( {\text{BT}} \right) \, = \frac{{{\text{BT}} \times {\text{FR}} \times C_{{{\text{H}}_{ 2} {\text{S}} }}^{\text{in}} \times 10^{ - 6} }}{{V_{\text{mol }} \times W}},$$

where BT is the breakthrough time, the time (min) when the H_2_S concentration reached 1 % (i.e., 15 ppmv), FR is the flow rate (mL min^−1^), *V*_mol_ is the molar volume (24.4 mL mol^−1^ at STP), *W* is the weight of the sorbent (in grams), and $C_{{{\text{H}}_{2} {\text{S}}}}^{\text{in}}$ is the initial concentration of the H_2_S in test gas mixture, respectively.

## Results and Discussion

### Morphology and Structural Characterization

**Fig. 2:**
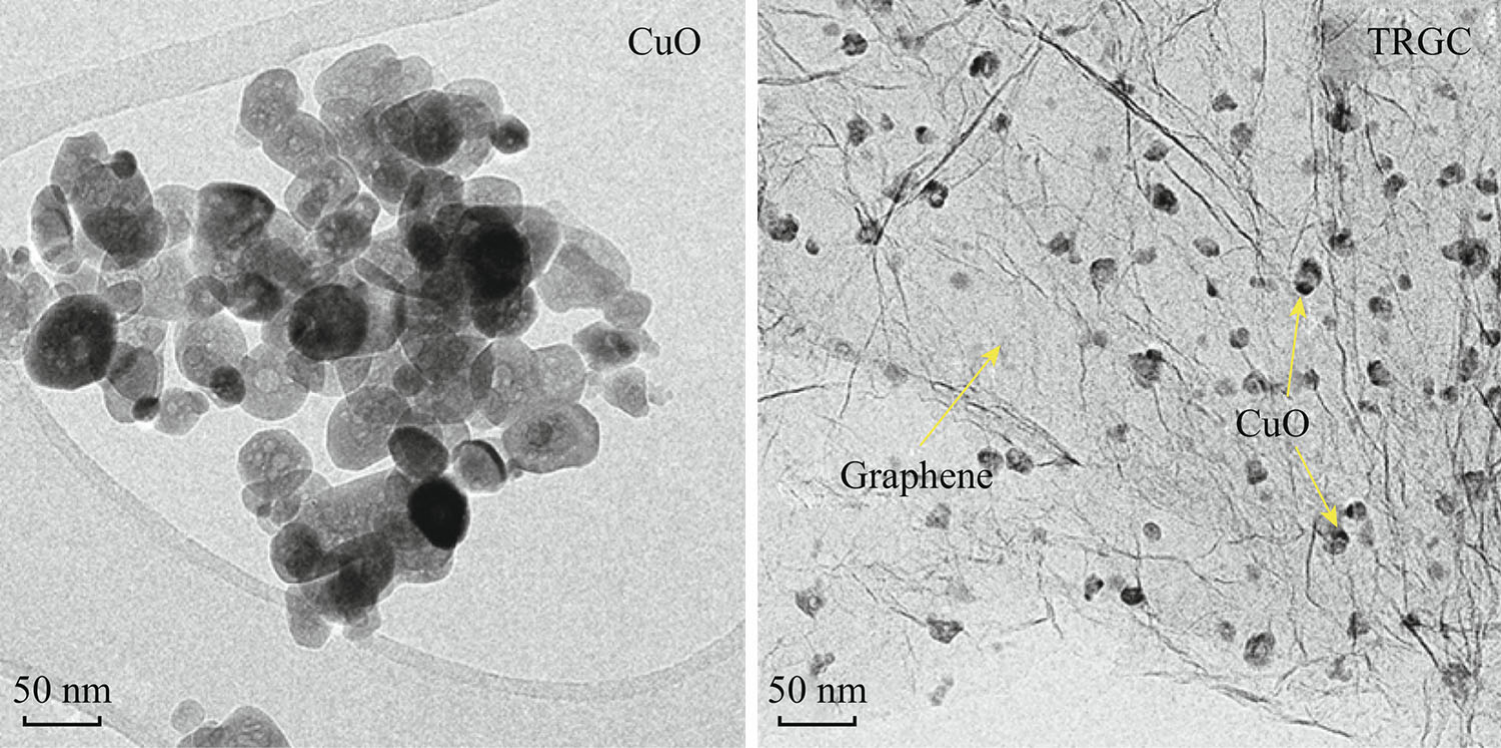
TEM images of CuO nanoparticles and TRGC composite

TEM images of CuO nanoparticles and TRGC composite are shown in Fig. 2. Thermal annealing of Cu(OH)_2_ under controlled conditions resulted into egg-shaped CuO nanoparticles with average size below 100 nm. A substantial decrease in the size of CuO particles (sub-20 nm) was noted in the presence of graphene which confirmed graphene’s control on the size and dispersion of the CuO seeds. Moreover, CuO nanoparticles were observed to be uniformly dispersed on the surface of graphene. Thus, the TEM images revealed that the nanostructured TRGC composites with a uniform CuO dispersion were successfully prepared.

**Fig. 3:**
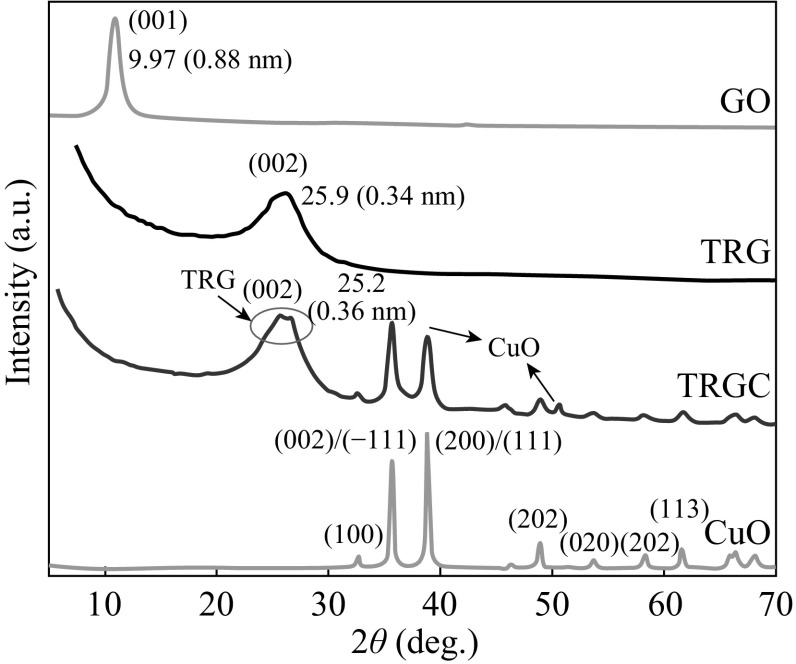
XRD diffractograms of GO, CuO, TRG, and TRGC

The structural features of the TRGC hybrid were elucidated using X-diffraction analysis. Figure 3 shows the XRD patterns of the CuO, GO, TRG, and TRGC hybrid. The XRD profile of GO exhibited characteristic peak at 9.7° corresponding to the (001) plane of GO. After thermal exfoliation of GO to TRG, the diffraction peak at 9.7° disappeared and a new broad peak at 25.9° was observed, which corresponded to the graphene (002) planes. The XRD pattern of CuO indicated the characteristic diffraction peaks primarily indexed to a monoclinic structure (JCPDS No. 96-410-5686). For TRGC hybrid, the diffraction pattern exhibited the presence of diffraction peaks from CuO nanoparticles and a new broad peak at 2*θ* = 24.33° corresponding to the (002) peak of thermally reduced graphite oxide [37]. Moreover, a (002) diffraction peak broadening and a shift toward lower 2*θ* values compared to the as-prepared pristine TRG signified the intercalation of CuO nanoparticles into TRG layers. In addition, no characteristic peaks corresponding to graphite oxide (the characteristic peak at around 2*θ* 9.7°) were observed in TRGC composite, indicating the successful thermal reduction of the layered GO in the composite. Hence, XRD revealed that the TRGC synthesis process involved the simultaneous thermal reduction of GO to TRG and in situ CuO nanoparticles formation.

**Fig. 4:**
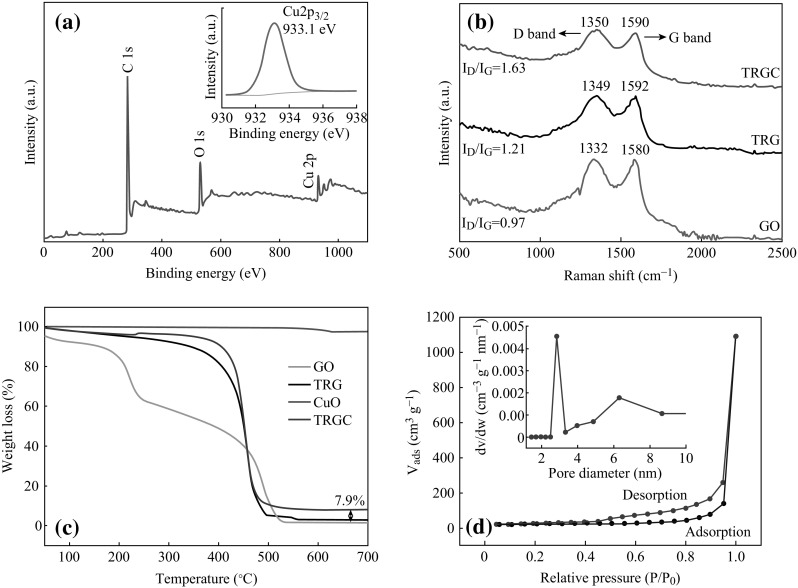
**a** XPS survey spectrum of TRGC (*inset* deconvolution of Cu 2P2), **b** Raman spectra of GO, TRG, and TRGC nanohybrid, **c** TGA curves of GO, TRG, CuO NPs, and TRGC composite, and **d** N_2_ adsorption–desorption isotherm and BJH pore size distribution plot (*inset*) of TRGC nanohybrid

XPS was used to trace the variations in surface composition after thermal reduction of GO and its interaction with in situ formed CuO nano-assemblies. Figure 4a shows the C1*s* and O1*s* chemical states of TRGC hybrid. The survey spectrum also shows the characteristic peaks of Cu2*p* and peaks of other elements were absent which ensured the contamination free in situ growth of CuO nano-assemblies on TRG sheets. The high-resolution scan of Cu 2*p*, as shown in Fig. 4a (inset), identified the exact peak location of Cu 2*p*_3/2_ at 933.1 eV. Hence, successful formation graphene/CuO nanohybrid under one-step thermo-annealed process was further conformed. The structural features of the TRGC composite were further elucidated by Raman spectroscopy. Generally, graphitic materials show the characteristic *D* and *G* bands corresponding to k-point phonons of *A*_1g_ symmetry and *E*_2g_ phonon of *sp* ^2^ carbon which are assigned to local defects and disorder especially at the edges of graphene and graphite platelets [38, 39]. Figure 4b shows the Raman spectra of the GO, TRG, and the corresponding TRGC hybrid. The pristine GO exhibited *G* and *D* bands at Raman shifts of 1580 and 1332 cm^−1^, respectively, with an intensity ratio, *I*_D_/*I*_G_ = 0.97. These two bands shifted to 1592 and 1349 cm^−1^ after the thermal treatment of GO. Such shifting is attributed due to the significant conversion of *sp* ^3^ to *sp* ^2^ carbon after thermal reduction. Also, trivial increase in *I*_D_/*I*_G_ to 1.21 was observed which indicated a decrease in the size of the in-plane *sp* ^2^ domains, which was mainly attributed to the removal of the oxygen functional group in GO during thermal reduction process [40]. For TRGC composite, further increase in *I*_D_/*I*_G_ = 1.63 ratio was observed which signified the simultaneous GO reduction and CuO nanoparticle formation implying successful synthesis of TRGC hybrid.

The thermal stability and the composition of the TRGC nanocomposite were further investigated by using TGA in air atmosphere. Figure 4c shows the thermal behavior of the TRGC nanocomposite and TRG, GO, and CuO nanoparticles, respectively. TGA curve of GO indicated two main weight loss steps, the first of which at around 100–250 °C was attributed to the decomposition of oxygen-containing functional groups on GO to CO, CO_2_, and H_2_O. The second degradation at around 400–600 °C was from the thermal decomposition of the GO structure. On the other hand, TRG thermogram indicated only one weight loss step. Slow decrease in weight between room temperature and 400 °C was attributed to the loss of surface bound water (50–250 °C) and the detachment of the oxygen functional groups (starting at 250 °C). It was followed by accelerated weight loss between 400 and 500 °C due to the oxidative degradation of the graphene carbon framework in addition to the removal of residual oxygen functionalities. The deposition of CuO nanoparticles on the surface of TRG exhibited similar degradation profile as TRG with enhancement in the onset of weight loss probably due to the protective layer of CuO preventing oxidative degradation of TRG. The CuO nanoparticles exhibited high thermal stability, with almost no obvious mass loss up to 700 °C. The final weight percentage of CuO in TRGC composite was measured to be at 7.9 wt%. Further, specific surface area and the pore size distributions of the as-prepared TRG and TRGC were measured using the BET and BJH methods (Fig. 4d). TRGC exhibited type IV isotherm with H1 and H2 hysteresis loops. The hysteresis loop in the relative pressure (*p*/*p*_0_) range of 0.4–0.9 is the characteristic of mesoporous materials.

The measured BET surface area of TRGC was 385 m^2^ g^−1^, which was higher than that of the as-prepared TRG (305 m^2^ g^−1^) suggesting that the in situ generation of CuO NPs effectively prevented overlap and coalescence of the graphene sheets. Moreover, the pore size of the TRGC was mainly distributed from 2.5 to 3.5 nm (Fig. 4d inset), which confirmed the nano-porous nature of the hybrid material.

### H_2_S Adsorption Breakthrough Tests

**Fig. 5:**
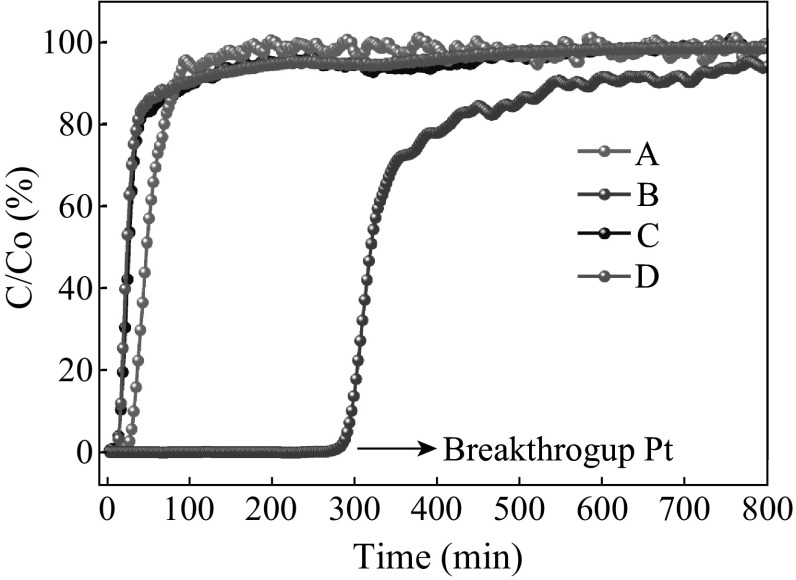
H_2_S breakthrough curves for TRG (**a**), TRGC (**b**), CH_4_ and CO_2_ breakthrough curves for TRGC (**c** and **d**)

TRGC-nanostructured hybrid was evaluated for removal of H_2_S at room temperature (30 °C) in the presence of CO_2_ and CH_4_. A dynamic H_2_S breakthrough test was used (Fig. 1) and the resulting breakthrough curves for tested adsorbents are shown in Fig. 5. Firstly, to gain insights about the contribution of CH_4_ sorption on the material, CH_4_ breakthrough curve of TRGC was measured under the same conditions mentioned above (Fig. 5). Saturation of the sorbent bed was completed in only 10 min, which indicated that CH_4_ adsorption on the adsorbent was negligible. Pure TRG exhibited negligible breakthrough capacity at 0.06 mmol H_2_S/g-sorbent and sulfur capacity of 30 mg S/100 g-sorbent due to the absence of any active sites. Remarkable difference in H_2_S breakthrough curves was observed for the TRGC hybrid composite in comparison with pristine graphene. TRGC sorbent exhibited H_2_S breakthrough point after 270 min which corresponded to the breakthrough capacity of 1.5 mmol H_2_S/g-sorb and sulfur capacity 3 g S/100 g-sorbent. The obtained capacities were significantly higher than pristine CuO sorbent (0.5 mmol/H_2_S/g-sorb) and other CuO-based sorbents studied under nearly identical conditions [41, 42]. Moreover, it is to be noted that the observed capacity value was for CuO loading at 7.9 wt% which is considerably lesser amount of the active component compared with other copper oxide-based desulfurization sorbents [43–45]. Also, the TRGC sorbent did not show any reactivity with CO_2_.

**Fig. 6:**
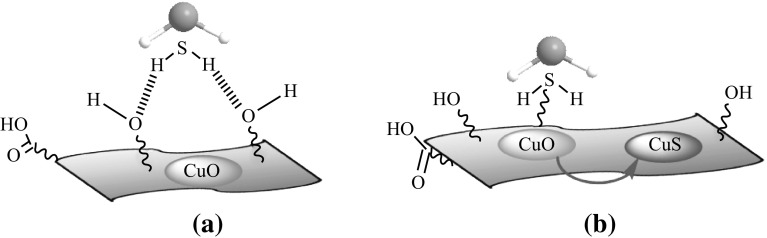
Schematic representation for possible H_2_S adsorption on TRGZ sorbent

The high H_2_S sorption capacity for TRGC sorbent is attributed due to the sub-20 nm CuO particles which enhanced the reactivity with H_2_S at low temperature in conjunction with the high surface area of TRG which supported more active sites, i.e., finely distributed CuO nanoparticles. As mentioned earlier, the nanosized grains can enhance the reactivity with H_2_S at low temperature and high surface areas can provide more active sites. Both properties are highly beneficial in improving the overall desulfurization performance of the sorbent [46]. The highly specific reaction of nanosized CuO toward H_2_S even under oxygen-depleted environment is the formation of CuS [47]. Further, the residual oxygen-containing functional groups on the basal planes of TRG (as confirmed by XPS) played a critical role in promoting oxygen activation by accelerating the electron transfer, thereby promoting the activity of the terminal groups in surface reaction  [48]. In addition, these functional groups helped the distribution of active CuO particles on the surface. So, the possible mechanism involves the initial physisorption of H_2_S molecules by oxygenated functional groups on the graphene surface (Fig. 6a) which later reach to the finely dispersed active CuO nanoparticles and get chemisorbed through reactive adsorption and converted into CuS (Fig. 6b) [49]. In present work, sorbent TRGC had a considerably large surface area (385 m^2^ g^−1^), which was almost 10 times that of CuO. Moreover, the pore volume of TRGC (1.6 cm^3^ g^−1^) was higher than that of CuO (0.083 cm^3^ g^−1^). These parameters also helped to synergistically enhance the H_2_S adsorption capacity of the TRGC adsorbent.

**Fig. 7:**
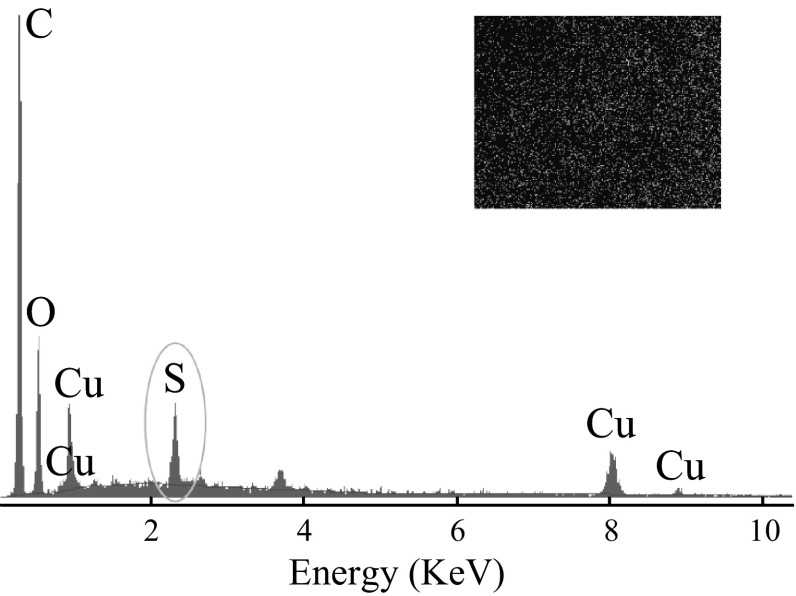
EDX spectra of TRGC and sulfur elemental mapping (*inset*) after H_2_S adsorption

Further, the EDX spectra of H_2_S-treated TRGC composites were recorded to monitor the changes in elemental composition (Fig. 7). Strong peak for sulfur element was observed indicating the reactive adsorption of H_2_S on the sorbent. The quantitative results of the S/Cu ratio were calculated from the area of the corresponding spectral *K* lines, and the amount of S in the composites was observed to be ~10 wt% which was in agreement with the breakthrough calculations. The inset sulfur element mapping image also confirmed the reactivity of the uniformly dispersed reactive CuO. However, to further elaborate this study, it would be important to investigate the reactivity of these adsorbents at higher temperatures, which is under investigation.

## Conclusions

In summary, a facile one-pot method for the synthesis of a graphene/CuO (TRGC) nanocomposite based on in situ thermal reduction of GO and simultaneous CuO nanoparticle synthesis is reported. Sub-20 nm CuO nanoparticles could be homogeneously dispersed on the surface of graphene platelets, which ensured the high-sulfur sorption capacity at ambient temperatures and at lower CuO loading. The as-prepared TRGC nanocomposite, generated by greener and efficient synthesis method, holds significant promise for potential applications in environment and energy sectors.
